# Long-term risk of major adverse cardiovascular events following ischemic stroke or TIA

**DOI:** 10.1038/s41598-023-35601-x

**Published:** 2023-05-23

**Authors:** Andreas Carlsson, Anna-Lotta Irewall, Anna Graipe, Anders Ulvenstam, Thomas Mooe, Joachim Ögren

**Affiliations:** Department of Public Health and Clinical Medicine, Östersund, Umeå University, Umeå, Sweden

**Keywords:** Risk factors, Disease-free survival, Stroke

## Abstract

Data are scarce on long-term outcomes after ischemic stroke (IS) or transient ischemic attack (TIA). In this prospective cohort study, we examined the cumulative incidence of major adverse cardiovascular events (MACE) after IS and TIA using a competing risk model and factors associated with new events using a Cox-proportional hazard regression model. All patients discharged alive from Östersund Hospital with IS or TIA between 2010 and 2013 (n = 1535) were followed until 31 December 2017. The primary endpoint was a composite of IS, type 1 acute myocardial infarction (AMI), and cardiovascular (CV) death. Secondary endpoints were the individual components of the primary endpoint, in all patients and separated in IS and TIA subgroups. The cumulative incidence of MACE (median follow-up: 4.4 years) was 12.8% (95% CI: 11.2–14.6) within 1 year after discharge and 35.6% (95% CI: 31.8–39.4) by the end of follow-up. The risk of MACE and CV death was significantly increased in IS compared to TIA (p-values < 0.05), but not the risk of IS or type 1 AMI. Age, kidney failure, prior IS, prior AMI, congestive heart failure, atrial fibrillation, and impaired functional status, were associated with an increased risk of MACE. The risk of recurring events after IS and TIA is high. IS patients have a higher risk of MACE and CV death than TIA patients.

## Introduction

Stroke fatality and the incidence of recurrent ischemic stroke are decreasing, but stroke remains a leading cause of death and disability worldwide^1,2^. In addition, those who survive the acute phase are still at high risk of recurrent cardiovascular (CV) events^3–8^. The risk of CV events seems to be almost equally high after the acute phase of a transient ischemic attack (TIA)^3^. In a meta-analysis from 2011^5^, the incidence of recurrent ischemic stroke (IS) was 11.1% and 26.4% at 1 year and 5 year, whereas more recent studies have reported an incidence of 3.6–6.0% and 9.5–16.0% at corresponding time points^9–14^. The 1-year incidence of acute myocardial infarction (AMI) has been reported between 0.4 and 2.6%^12,15,16^. Long-term data on the overall risk of new CV events after discharge in an unselected ischemic stroke (IS) and TIA population are scarce. As the population in need of secondary preventive measures grows as a consequence of improved survival, up-to-date knowledge on the long-term risk of new events and associated risk factors is crucial. This study aimed to: (1) assess the cumulative incidence of major adverse cardiovascular events (MACE), defined as the composite of IS, type 1 acute myocardial infarction (AMI), and CV death, after IS and TIA, (2) compare the incidence of MACE between IS and TIA survivors and (3) identify factors associated with an increased risk of an event.

## Methods

### Study population

This prospective cohort study included stroke and TIA patients identified as part of the Nurse-based Age-independent Intervention to Limit Evolution of Disease (NAILED) trial. The NAILED trial was a randomized controlled trial (RCT) performed at Östersund Hospital to investigate whether telephone-based follow-up after stroke, TIA, myocardial infarction, or unstable angina can improve CV outcomes compared to usual care^17,18^. Between January 1, 2010, and December 31, 2013, all patients admitted to Östersund Hospital with stroke or TIA were screened for participation in the NAILED trial. Östersund Hospital is the only hospital in the county of Jämtland. All patients, except those in terminal care, who experience symptoms of suspected stroke or TIA are referred there. During the screening phase of the NAILED trial, hospital records of all patients who had undergone brain computed tomography (CT) scans were reviewed daily to identify patients who were subsequently diagnosed with an acute IS or TIA. In addition, all patients in the stroke unit were checked in order to not miss any stroke or TIA patient who was diagnosed without undergoing a CT scan. All identified stroke and TIA patients who survived through hospitalization qualified for inclusion in the present study.

### Endpoints

The primary endpoint, MACE, was defined as the composite of IS, type 1 AMI, and CV death, whichever happened first. Deaths were classified as CV, non-CV, and unknown cause of death. A stroke or AMI followed by death within 30 days was considered fatal and included in the primary outcome as CV death. Death due to sudden cardiac death, congestive heart failure, cardiovascular procedure, cardiovascular bleeding, and pulmonary embolism was also classified as CV deaths^19^. The definition of IS was an acute episode of focal or global cerebral, spinal, or retinal dysfunction caused by infarction of central nervous system tissue^20^. Episodes of focal cerebral dysfunction without evidence of brain infarction and with symptoms resolving within 24 h were counted as TIA. Episodes of focal cerebral dysfunction with evidence of brain infarction and with symptoms resolving within 24 h were counted as Stroke. The secondary endpoints were defined as the individual components of the primary endpoint. AMI was defined in accordance with the third universal definition of AMI, but only type 1 AMIs were included. A type 1 AMI was defined as an AMI typically caused by plaque rupture, ulceration, fissuring, erosion, or dissection, resulting in an intraluminal thrombus and decreased myocardial blood flow^21^.

### Data collection

Baseline data were collected during the screening phase of the NAILED trial through patient interviews and reviews of the medical records. Included patients were followed for recurring events from discharge until December 31, 2017. Patients who moved were censored from the outcome analysis at the date they moved. CV events were identified through a structured review of discharge records for hospitalizations at the medical department of Östersund Hospital and through complementary screening of the in-patient register to catch events occurring in other hospital departments. Four experienced medical doctors (3 consultants and 1 senior resident), all of whom were part of the study team, adjudicated identified events based on the information available in the medical records. Death certificates and patient records were used to adjudicate causes of death. Each doctor worked separately using a standardized workflow algorithm. Endpoints were adjudicated in accordance with pre-specified endpoint definitions. Definitions of outcome events are described in the supplementary methods.

### Statistical analysis

Baseline characteristics are presented as median values with interquartile ranges (IQRs) for continuous variables and as proportions (percentages) for categorical variables. The number of cases with missing data was small and reported for each variable separately. Due to an expectation of high all-cause mortality in this population during long-term follow-up, cumulative incidence was calculated using a competing risk model according to Fine-Gray with death as a competing event.

Cox proportional hazards regression was used to identify possible factors associated with MACE. All variables with p < 0.10 in univariable Cox regression analyses, age, and sex was included in a multivariable model. We reduced the model stepwise by excluding the least significant variable manually until only significant variables remained. Sex and age were retained regardless of significance. The final multivariable analysis was based on complete cases with no missing data. Results are presented as hazard ratios (HRs) with 95% confidence intervals (CIs). p-values < 0.05 were considered significant.

SPSS version 27.0 and SAS 9.4 were used to perform Cox regression analyses, Kaplan–Meier estimates, and to determine cumulative incidences considering a competing risk assumption.

### Ethics

This is an observational study; no interventions took place that affected patient care. The NAILED trial was approved by the Regional Ethical Review Board, Umeå, Sweden, on October 28, 2009 (Dnr 09-142M). Studies of baseline characteristics and follow-up regarding new events among participants not included for randomization in the NAILED trial were approved on June 10, 2013 (Dnr 2013-204-32M), and an extended follow-up period was approved on January 13, 2014 (Dnr: 2014-416-32). The study was performed in accordance with the Declaration of Helsinki and all participants provided informed written consent of participation.

## Results

### Baseline characteristics

**Figure 1:**
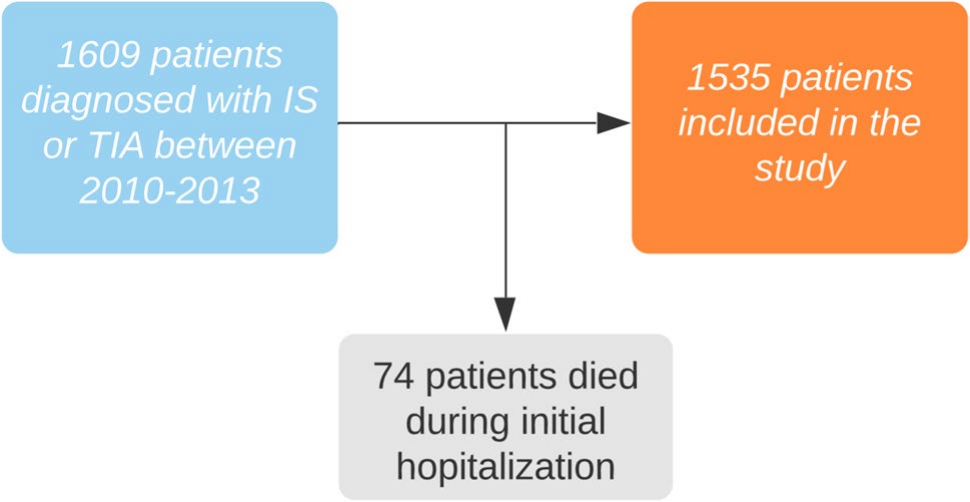
Flowchart of patient inclusion.

A total of 1535 patients were included in the study (Fig. 1). The index event was IS in 71% and TIA in 29% of cases. The median age was 77 years (75 years for men, 79 years for women) and 55.6% were men. Baseline characteristics are given in Table 1. By the end of follow-up, 684 patients had died (44.6% of the study population, 49.2% and 33.3% in the IS and TIA subgroup, respectively), including 225 who died during the first year after discharge. The cause of death was classified as CV death in 43.8% of the deaths in the IS group and 31.1% of the deaths in the TIA group (Supplementary Table S1).

**Table 1:** Baseline patient characteristics among 1535 patients discharged after hospitalization for ischemic stroke or TIA.

Variable	IS and TIA (n = 1535)	IS (n = 1090)	TIA (n = 445)
Median age, years (IQR)	77 (66–89)	78 (67–89)	74 (63–86)
Men	854 (55.6)	609 (55.9)	245 (55.1)
Current smoking*	192 (12.7)	138 (13.0)	54 (12.1)
Previous smoking*	522 (34.7)	364 (34.2)	158 (35.7)
mRS 0–2 at discharge^†^	1001 (65.4)	595 (54.8)	406 (91.2)
Median BMI, kg/m^2^ (IQR)^‡^	25.4 (23.1–28.5)	25.3 (22.8–28.7)	25.8 (23.4–28.3)
Comorbidities			
Hypertension	988 (64.4)	743 (68.2)	245 (55.1)
Congestive heart failure	128 (8.3)	104 (9.5)	24 (5.4)
Diabetes mellitus	302 (19.7)	233 (21.4)	69 (15.5)
Atrial fibrillation	283 (18.4)	219 (20.1)	64 (14.4)
GFR, < 60 ml/min	590 (38.4)	432 (39.6)	158 (35.5)
Medical history			
Prior AMI^§^	181 (11.8)	139 (12.8)	42 (9.4)
Prior IS	267 (17.4)	217 (19.9)	50 (11.2)
Prior hemorrhagic stroke	21 (1.4)	16 (1.5)	5 (1.1)
Prior TIA	92 (6.0)	56 (5.1)	36 (8.1)
Medication at discharge			
Aspirin	656 (42.7)	455 (41.7)	201 (45.2)
Clopidogrel	610 (39.7)	437 (40.1)	173 (38.9)
Warfarin	241 (15.7)	176 (16.1)	65 (14.6)
Statin	938 (61.1)	650 (59.6)	288 (64.7)
Beta-blocker	684 (44.6)	521 (47.8)	163 (36.6)
ACE inhibitor	471 (30.7)	361 (33.1)	110 (24.7)
AT2 antagonist	230 (15.0)	165 (15.1)	65 (14.6)
Calcium channel blockers	389 (25.3)	287 (26.3)	102 (22.9)
Diuretics	539 (35.1)	408 (37.4)	131 (29.4)

Values are given as n (%) unless otherwise noted.

*IS* ischemic stroke, *TIA* transient ischemic attack, *IQR* interquartile range, *mRS* modified Rankin scale, *BMI* body mass index, *GFR* glomerular filtration rate, *AMI* acute myocardial infarction, *ACE* angiotensin converting enzyme, *AT2* angiotensin receptor type 2.

*Missing values for 29 participants.

^†^Missing values for 4 participants.

^‡^Missing values for 30 participants.

^§^Missing values for 3 participants.

### Risk of MACEs

**Figure 2:**
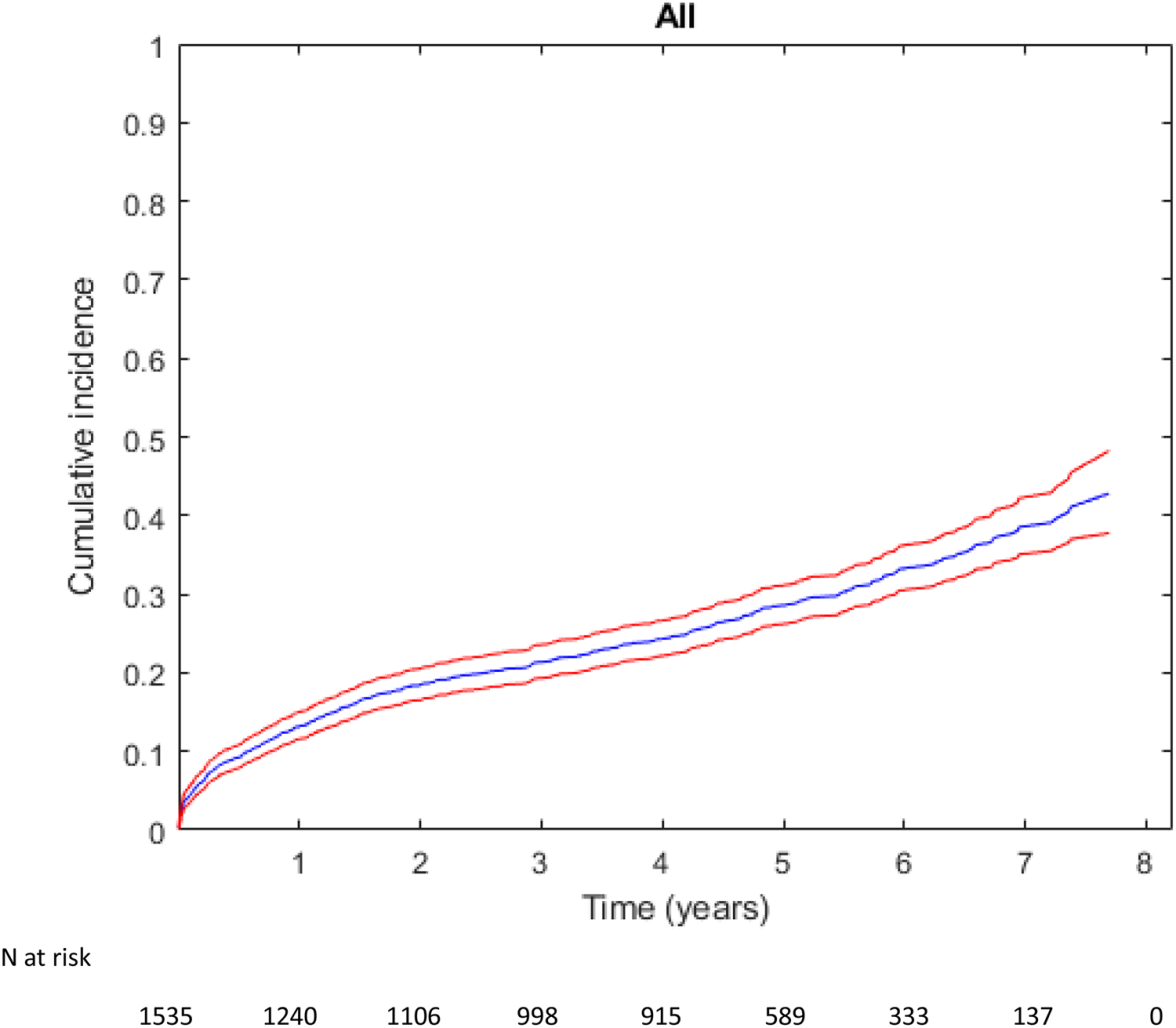
Cumulative incidence of MACE among 1535 patients discharged after hospitalization for ischemic stroke or TIA between January 1, 2010, and December 31, 2013, and followed until December 31, 2017. Red lines indicate 95% confidence interval.

**Figure 3:**
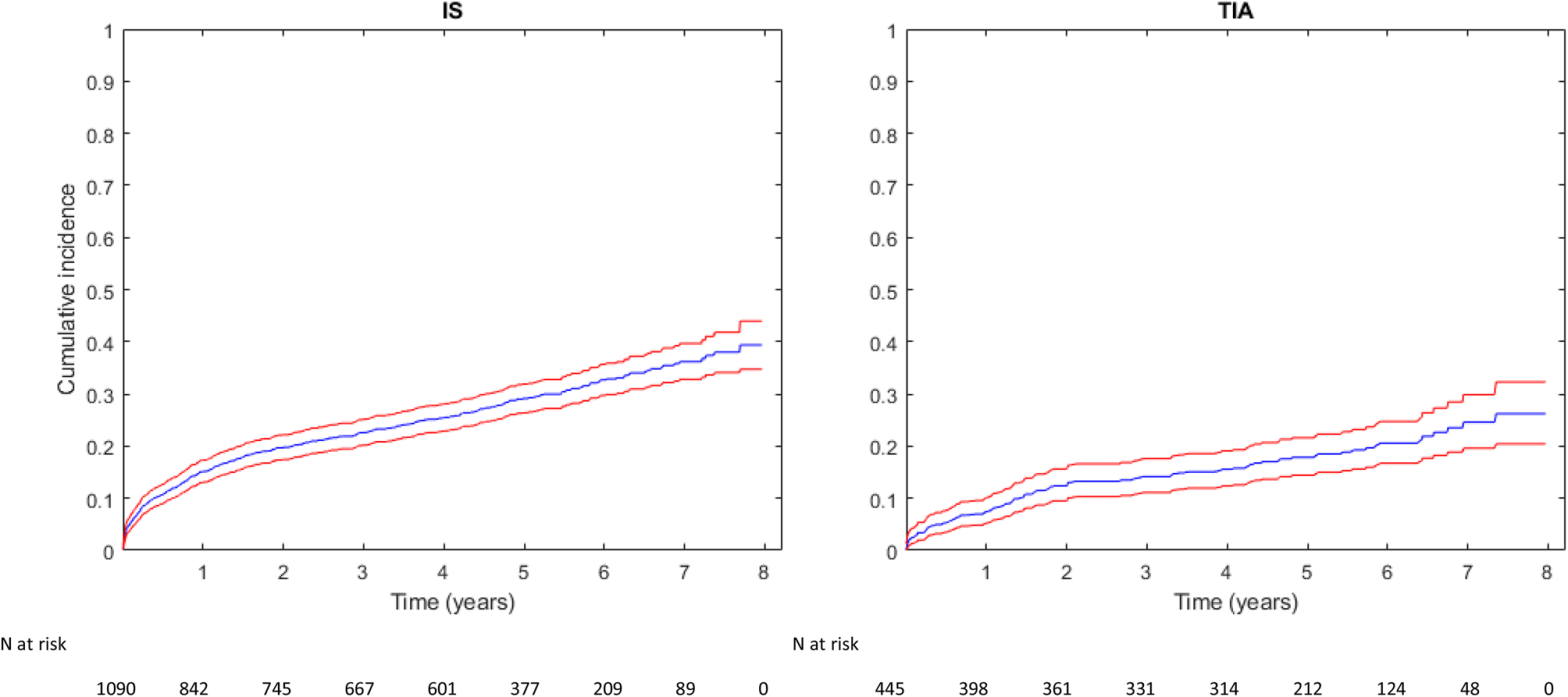
Cumulative incidence estimate of MACE stratified by index event among 1535 patients discharged after hospitalization for ischemic stroke or TIA between January 1, 2010, and December 31, 2013, and followed until December 31, 2017.

Cumulative incidences for the primary and secondary endpoints after 1 year and at the end of the follow-up period are presented in Table 2, and the number of events contributing to the composite primary endpoint is provided in Supplementary Table S2. During a median follow-up of 4.4 years, 440 patients (28.7%) reached the primary endpoint [188 (42.7%) were IS, 42 (9.5%) type 1 AMI, and 210 (47.7%) CV deaths]. The cumulative incidence of MACE was 12.8% (95% CI: 11.2**–**14.6) within 1 year after discharge and 35.6% (95% CI: 31.8–39.4) by the end of follow-up (Table 2 and Fig. 2). The cumulative incidence of MACE was significantly higher for IS patients than TIA patients at both 1 year and the end of follow-up (p < 0.001; Table 2 and Fig. 3).

**Table 2:** Number of cases and cumulative incidence estimate per endpoint for 1535 patients discharged after hospitalization for ischemic stroke or TIA between January 1, 2010, and December 31, 2013, and followed until 1 year after index event and December 31, 2017.

Endpoint	Cumulative incidence, 1 year (95% CI)	n (% of total)	Cumulative incidence, end of follow-up (95% CI)	n (% of total)
Index event: IS and TIA				
MACE	12.8 (11.2–14.6)	197 (12.8)	35.6 (31.8–39.4)	440 (28.7)
IS	5.9 (4.8–7.1)	89 (5.8)	14.0 (12.2–16.0)	193 (12.6)
Type 1 AMI	1.0 (0.6–1.6)	14 (0.9)	4.1 (3.0–5.5)	49 (3.2)
CV death	7.0 (5.8–8.4)	107 (7.0)	25.3 (21.8–29.0)	281 (18.3)
Index event: IS				
MACE	15.1 (13.0–17.3)	164 (15.0)	39.4 (34.8–44.0)	349 (32.0)
IS	6.0 (4.7–7.5)	65 (6.0)	14.3 (12.1–16.6)	139 (12.8)
Type 1 AMI	0.8 (0.4–1.5)	9 (2.0)	4.8 (3.3–6.6)	39 (3.6)
CV death	9.3 (7.6–11.1)	101 (9.8)	28.9 (24.6–33.4)	235 (21.6)
Index event: TIA				
MACE	7.4 (5.2–10.1)	33 (7.4)	26.2 (20.5–32.3)	91 (20.4)
IS	5.6 (3.7–8.0)	24 (5.4)	13.3 (10.2–17.0)	54 (12.1)
Type 1 AMI	1.4 (0.6–2.8)	5 (1.1)	2.5 (1.3–4.3)	10 (2.2)
CV death	1.6 (0.7–3.1)	6 (1.3)	16.2 (11.2–22.1)	46 (10.3)

*IS* ischemic stroke, *TIA* transient ischemic attack, *MACE* major adverse cardiovascular event, *AMI* acute myocardial infarction, *CV* cardiovascular.

For the individual components of the primary endpoint, the cumulative incidence at the end of follow-up was 14.0% for IS (95% CI: 12.2–16.0, n = 193), 4.1% for AMI (95% CI: 3.0–5.5, n = 49), and 25.3% for CV death (95% CI: 21.8–29.0, n = 281). Out of 107 cases of CV death within the first year after discharge, 68% occurred within 90 days. The incidence of CV death was significantly higher for IS patients than TIA patients at both 1 year and end of follow-up (p < 0.001), Table 2. No significant difference in IS or type 1 AMI was found between stroke and TIA patients (p = 0.8145 and p = 0.2438, respectively). The 1-year and end of follow-up cumulative incidences of MACE, CV death, IS, and AMI in the IS and TIA patient groups are provided in Table 2 and Fig. 3.

### Predictors

In a multivariable Cox regression analysis, age, GFR < 60 ml/min, prior IS, prior AMI, prior CHF, atrial fibrillation, mRS ≥ 3 at discharge, and IS compared to TIA as the index event were independently associated with an increased risk of MACE during the study period. Hazard ratios for all predictors are presented in Table 3 and univariable analyses are presented in Supplementary Table S3. Diabetes, hypertension, antiplatelet medication at discharge, and statins at discharge had a p-value < 0.10 in the univariate analysis but were not significant in the multivariate analysis and therefore excluded in the final multivariate model.

**Table 3:** Multivariate Cox regression analysis of predictors of major adverse cardiovascular events after ischemic stroke or TIA among 1535 patients.

Variable	HR (95% CI)	P-value
Age (per year)	1.06 (1.04–1.07)	< 0.001
Female sex	0.87 (0.71–1.06)	0.175
GFR < 60	1.30 (1.03–1.63)	0.025
Prior IS	1.46 (1.17–1.83)	< 0.001
Prior AMI	1.36 (1.05–1.78)	0.022
Prior CHF	1.44 (1.07–1.95)	0.013
Atrial fibrillation	1.31 (1.06–1.61)	0.007
mRS ≥ 3 at discharge	1.33 (1.08–1.65)	0.009
IS as index event	1.32 (1.03–1.70)	0.029

*HR* hazard ratio, *GFR* glomerular filtration rate, *IS* ischemic stroke, *AMI* acute myocardial infarction, *CHF* congestive heart failure, *mRS* modified Rankin scale.

## Discussion

In this unselected IS and TIA population, the cumulative incidence of MACE after IS or TIA was 35.6% (95% CI: 31.8–39.4), with a cumulative incidence of 12.8% (95% CI: 11.2**–**14.6) within the first year. There was a significantly higher risk of MACE in IS patients than TIA patients. The difference was driven by a significant difference in risk of CV death, especially early during follow-up. We did not find a significant difference in risk of IS after discharge for IS or TIA. Well-known risk factors for CV events were associated with an increased risk of a MACE.

Compared to the general population, the long-term risk of MACE is elevated after an IS or TIA, with the highest risk during the first months following an event^3–8^. In addition, the mortality risk is higher within the first months after an IS^4,22,23^. Our results show the same epidemiological pattern regarding both early and long-term incidence. CV death was the most prevalent component of MACE in our study population, and the risk of CV death was significantly higher in the IS subgroup than in patients with a TIA, with most of the difference within the first months (Fig. 3). The proportion of non-CV death was similar in the IS and TIA subgroups; therefore, the all-cause mortality was higher in the IS subgroup (Supplementary Table S1). The IS patients were older, prior CV disease was more common, and they were more disabled at discharge according to the mRS. These factors are well-known risk factors for mortality after a cerebrovascular event^10,24,25^ and, not surprisingly, were all positively associated with MACE in our multivariate analysis. Age inevitably increases the risk of death. Age also entails an accumulation of known riskfactors of CV disease including hypertension, hyperlipidemia and hyperglycemia, impaired renal function, and prior CV disease. An increased risk of death could limit the effectiveness of secondary prevention, but evidence suggests effect of treatment with antihypertensives and lipid-lowering treatment irrespective of age and should not be withheld from the very elderly because of age itself^26–28^.

Though the risk of death differed between IS and TIA patients, we found that the risk of IS was similar in the two subgroups, and the risk of having an IS was greater than having an AMI. This is in accordance with previous studies^3,11,16,29^. A meta-analysis published in 2011 found an incidence of recurrent IS of 11.1% at 1 year and 26.4% at 5 years, whereas more recent studies reported an incidence in the range of 3.6–6.0% and 9.5–16.0% at the corresponding timepoints^9–14^. Our results are in line with the more recent studies. Comparisons between studies are difficult due to differences in populations, but the lower incidence rate is in line with data on temporal trends that suggest a decline in stroke recurrence^9,30^.

The 1-year incidence of AMI is similar to the findings in previous studies on AMI after IS, with incidences between 0.4 and 2.6%^12,15,16^. In addition, the risk during long-term follow-up is comparable; though the risk of an AMI is lower than the risk of an IS, but the risk of an AMI is increased compared to a population without stroke^3,16,31^. IS patients with an event of acute coronary syndrome have an increased risk of death compared with IS patients without^32^. The cumulative incidence was similar in the IS and TIA subgroups during the first year, but then the risk was increased in the IS subgroup (Table 2). However, the number of AMIs in this study and the numbers at risk in the last years of follow-up were small and, therefore, no conclusions can be drawn.

This study adds an updated estimate of the overall risk of CV events after an IS or TIA, as well as of the separate risk of IS and AMI and any differences in risk depending on the index event. The risk of IS and AMI remains relatively constant in both sub-groups after the first year during a long-term follow-up, which underlines the importance of long-term follow-up and prevention in patients who have had an IS or TIA. During the 2000s, the mortality after an IS and the risk of recurrent events decreased^9,30,33,34^, but the question remains as to whether there are strategies to continue to improve short- and long-term outcomes. Reperfusion treatments, such as thrombolysis and thrombectomy, may contribute to a decrease in mortality and improve functional outcomes after stroke, but are only available for a small part of the population. Moreover, the effect on long-term outcomes in an unselected population remains to be studied^35,36^. Other future research questions include how to improve prevention of new MACE. Based on modifiable risk factor levels, previous studies have indicated missed opportunities in secondary prevention of CV events. More than half of the patients in this IS and TIA population were included in a RCT that investigated whether a nurse-led and telephone-based follow-up strategy could decrease MACE in a population after IS, TIA, or acute coronary syndrome. It showed that the intervention improved not only blood pressure and lipid levels^37^, but also decreased recurrence of MACE, suggesting that a structured long-term follow-up of modifiable risk factors could lead to fewer MACE^38^.

### Strengths and limitations

This study investigated the long-term risk of MACE after IS or TIA in an unselected, single-center cohort. The population was screened for participation in the NAILED stroke trial, a registered RCT investigating whether telephone-based follow-up after stroke or TIA can improve CV outcomes compared to usual care. This current study was a non-registered secondary prospective analysis of the screening cohort. It included all patients with an IS or TIA treated at Östersund hospital 2010–2013, regardless of age or prior events. This may lead to higher event rates compared to studies that limited inclusion to first-time strokes. On the other hand, our population is more representative of the unselected population found in stroke wards in general practice. A limitation is the use of hospital records to identify new events. Events of minor stroke and TIA in patients who did not seek hospital following the event might therefore been missed. Another limitation is the relatively small number included in the study, which decreases the power, especially in the subgroup analysis. The single-center design may negatively affect the external validity. However, the population and stroke care during the study period is comparable to the care in other Swedish hospitals^39^. In addition, the 1-year incidence of recurrent IS in our population is comparable to other recent studies. A strength of the study is the adjudication process of all clinical endpoints, which should result in higher quality endpoint data compared to studies that rely on registers and diagnosis codes only. This also enabled distinction between type 1 and type 2 AMIs, which is a strength because they may have different prognostic impacts. Our study included only type 1 AMIs, which have led to lower event rates compared to register-based studies.

## Conclusions

Among unselected patients discharged after a stroke or TIA, 42.8% suffered a MACE during a median follow-up of 4.4 years. IS and CV death were by far the most prevalent components of the primary endpoint, and the risk of these events was particularly high early during follow-up. CV death was comparably less common in the subgroup with TIA, a difference that was at least partly related to age, comorbidity, and inherent differences in the severity of the index event. Importantly, the risk of IS was similar after TIA or stroke.

## Supplementary Information

Supplementary Information.

## Data Availability

As open access to individual-level data was not specified in the original application approved by the ethics committee, the underlying data is only available upon reasonable request. Please contact the corresponding author.

## Abbreviations

AMI: Acute myocardial infarction
CI: Confidence interval
CV: Cardiovascular
IQR: Interquartile range
MACE: Major adverse cardiovascular event
NAILED: Nurse-based, age-independent intervention to limit evolution of disease.
RCT: Randomized controlled trial
TIA: Transient ischemic attack
