# Supplementary material for: Long-term risk of major adverse cardiovascular events following ischemic stroke or TIA
Source: Sci Rep. 2023 May 23;13:8333. doi: 10.1038/s41598-023-35601-x (PMC10206105; doi:10.1038/s41598-023-35601-x)
Supplement: Supplementary file 1 — Supplementary Information. [file 41598_2023_35601_MOESM1_ESM.pdf]

SUPPLEMENTAL MATERIAL

Long-term risk of major adverse cardiovascular events following ischemic stroke or TIA

Andreas Carlsson, Anna-Lotta Irewall M.D PhD, Anna Graipe M.D PhD, Anders Ulvenstam M.D PhD, Thomas Mooe Prof, Joachim Ögren M.D PhD

Supplemental Table S1. Number of patients who died after discharge for an IS or TIA until December, 31, 2017.

Cause	IS	TIA	Total
CV death	235 (21.6)	46 (10.3)	281 (18.3)
Non-CV death	277 (25.4)	95 (21.3)	372 (24.2)
Undetermined	24 (2.2)	7 (1.6)	31 (2.0)
Total	536 (49.2)	148 (33.3)	684 (44.6)

Values are n (%).

Supplemental Table S2. Number of contributing events in the MACE composite presented in Table 2.

	**1 year**	**End of follow-up**
**Index event: IS and TIA**		
IS	84	170
Type 1 AMI	12	42
CV death	101	228
**Index event: IS**		
IS	61	121
Type 1 AMI	7	33
CV death	96	195
**Index event: TIA**		
IS	23	49
Type 1 AMI	5	9
CV death	5	33

IS: ischemic stroke; TIA: transient ischemic attack; MACE: major adverse cardiovascular event; AMI: acute myocardial infarction; CV: cardiovascular.

Supplemental Table S3. Univariate Cox regression analysis of predictors of major adverse cardiovascular events after ischemic stroke or TIA among 1535 patients.

**Variable**	**HR (95% CI)**	**P-value**
Age	1.075 (1.064 - 1.087)	<0.001
Female sex	0.903 (0.749 - 1.089)	0.285
Diabetes mellitus	1.286 (1.026 - 1.611)	0.029
Insulin-dependent DM	1.383 (1.049 - 1.824)	0.022
Current or prior smoker	1.053 (0.870 - 1.274)	0.599
GFR <60	2.660 (2.197 - 3.220)	<0.001
Hypertension	1.638 (1.329 - 2.019)	<0.001
Prior IS	2.014 (1.628 - 2.492)	<0.001
Prior TIA	1.053 (0.708 - 1.565)	0.799
Prior ICH	1.737 (0.897 - 3.361)	0.101
Prior AMI	2.038 (1.598 - 2.598)	<0.001
Prior CHF	2.925 (2.230 - 3.837)	<0.001
Atrial fibrillation	2.037 (1.674 - 2.479)	<0.001
MRS 3-5 at discharge	2.405 (1.991 - 2.905)	<0.001
Warfarin at discharge	1.167 (0.916 - 1.488)	0.210
Antiplatelet medication at discharge	0.773 (0.618 - 0.966)	0.024
Statin at discharge	0.545 (0.452 - 0.659)	<0.001
IS as index event	1.832 (1.454 - 2.307)	<0.001

HR: hazard ratio; CI: confidence interval; DM: diabetes mellitus; GFR: glomerular filtration rate; IS: ischemic stroke; TIA: transient ischemic attack; ICH: intracranial hemorrhage; AMI: acute myocardial infarction; CHF: congestive heart failure; mRS: modified Rankin scale.

Supplementary methods 1

Definition of outcome events

With minor modifications, the definitions found in this document are based on the Standardized Data Collection for Cardiovascular Trials Initiative (CDISC) draft "Standardized Definitions for Cardiovascular and Stroke Endpoint Events in Clinical Trials". The definition of myocardial infarction complies with the "2012 Third Universal Definition of Myocardial infarction".

Except for death and revascularisation, outcome events were identified based on a review of medical discharge records and registered discharge diagnoses. Consequently, potential events of myocardial infarction, stroke, or TIA required the patient to be admitted to hospital and hospitalised to be included as outcome events. Identification of revascularisation events was based on registry data, which included both in-patient and out-patient procedures performed at Östersund Hospital or the University Hospital of Northern Sweden. The patient medical record is electronic and connected with the National Civil Register. When a patient died within the national boarders of Sweden, this information, including the date of death, was usually available within 24 hours. All care givers within Jämtland-Härjedalen used the same electronic medical record. Thus, for events of death occurring within the county, all medical documentation except for forensic protocols was available to the reviewers. In a few cases (n=14), the underlying cause of death could not be classified as cardiovascular or non-cardiovascular due to absent or insufficient documentation.

Outcome events

1. **Death**
   Classified as cardiovascular, non-cardiovascular, or undetermined.

   A. **Cardiovascular death**
      Includes death due to any of the following conditions:

      A. Acute myocardial infarction
         As defined below or verified by autopsy. Includes death by any cardiovascular mechanism (arrhythmia, sudden cardiac death, congestive heart failure, stroke, pulmonary embolism, peripheral artery disease, invasive revascularisation procedure) within 30 days of an acute myocardial infarction.

      B. Sudden cardiac death
         Unexpected death, *not* caused by acute myocardial infarction. Includes death according to any of the following scenarios:
         i. Witnessed and occurring without new or worsening symptoms;
         ii. Witnessed within 60 minutes after onset or worsening of cardiac symptoms;
         iii. Witnessed or unwitnessed with arrhythmia identified by ECG recording, defibrillator monitoring, or implantable device such as cardioverter defibrillator or loop recorder;
         iv. Death after unsuccessful resuscitation from cardiac arrest;
         v. Death after successful resuscitation from cardiac arrest without identification of a specific cardiac or non-cardiac aetiology;
         vi. Patients found dead within 24 hours of last being seen well and stable and without signs of a specific non-cardiovascular cause.

C. Congestive heart failure
Death in association with clinical worsening of symptoms of congestive heart failure regardless of aetiology (ischaemic heart disease, non-ischaemic cardiomyopathy, valvular disease).

D. Stroke
Death as a direct consequence of stroke or indirectly due to a related complication.

E. Cardiovascular procedure
Death caused by immediate complication of a cardiac procedure.

F. Cardiovascular bleeding
Death due to any of the following:
i. Non-stroke intracerebral haemorrhage;
ii. Non-traumatic, non-procedural vascular rupture (e.g., aortic aneurysm);
iii. Cardiac tamponade.

G. Other
Death due to other cardiovascular conditions, such as pulmonary embolism or peripheral artery disease.

B. **Non-cardiovascular death**
Documentation supporting death due to a specific, non-cardiovascular cause, such as:
i. Pulmonary;
ii. Renal;
iii. Gastrointestinal;
iv. Pancreatic;
v. Infection (includes sepsis);
vi. Suicide;
vii. Trauma;
viii. Malignancy.

C. **Undetermined cause of death**
Death cannot be classified as cardiovascular or non-cardiovascular due to absent documentation.

2. Acute myocardial infarction
Based on 2012 Third Universal Definition of Myocardial Infarction. Acute myocardial infarction should be used when there is evidence of myocardial necrosis in the clinical setting of myocardial ischaemia, i.e., any of the following should apply:
i. Rise and/or fall of cardiac biomarkers with at least one measurement above the 99th percentile of the upper reference limit (URL) in combination with at least one of the following:
 a. Ischaemic symptoms;
 b. New or presumed new ECG changes (described in further detail below): ST-elevation, ST-depression, T-wave inversion, or LBBB;
 c. Development of pathological Q-wave (described in further detail below);
 d. Evidence by imaging of loss of viable myocardium or new regional wall motion abnormality;
 e. Intracoronary thrombus identified by coronary angiography or autopsy.

ii. Sudden cardiac death preceded by clinical symptoms of cardiac ischaemia *and* new onset ischaemic ECG changes in a patient in whom death occurred before cardiac biomarkers were obtained or would be increased.
iii. Percutaneous coronary intervention (PCI)-related MI: elevation of cardiac biomarkers >5 times the URL (or increased >20% in a patient with stable elevation or falling values) *and* at least one of the following:
 a. Ischaemic symptoms;
 b. Ischaemic ECG changes;

c. Angiographic evidence of procedural complication;
d. Evidence by imaging of loss of viable myocardium or new regional wall motion abnormality.

iv. Stent thrombosis detected by coronary angiography or autopsy in combination with ischaemic symptoms and a rise/fall of cardiac biomarkers with at least one value above the 99th percentile of the URL.

v. Coronary artery bypass grafting (CABG)-related MI: elevation of cardiac biomarkers (>10 times the URL) in a patient with normal baseline levels in combination with any of the following:
a. New Q-wave or LBBB;
b. Angiographic evidence of occlusion of a new graft or a native coronary artery;
c. Evidence by imaging of loss of viable myocardium or new regional wall motion abnormality.

Subclassification of myocardial infarction:

Type 1: Spontaneous myocardial infarction

Plaque rupture, ulceration, fissuring, erosion or dissection resulting in intraluminal thrombus and decreased myocardial blood flow.

Type 2: Myocardial infarction secondary to an ischaemic imbalance

A condition other than coronary artery disease contributes to an imbalance between myocardial oxygen demand and supply, resulting in myocardial necrosis.

Type 3: Myocardial infarction resulting in death when biomarker values are unavailable

Type 4a: Myocardial infarction related to PCI

Type 4b: Myocardial infarction related to stent thrombosis

Type 5: Myocardial infarction related to CABG

Criteria for ECG changes suggestive of ischaemia:

STEMI: *ST-elevation* in two contiguous leads with cut-points: 1 mV in all leads other than lead V2-V3 where the following cut points apply: 2 mV for men ≥40 years; 2·5 mV for men <40 years; 1·5 mm for women.

NSTEMI: *ST-depression* ≥0·05 mV in two contiguous leads or *T-wave inversion* ≥0·3 mV in two contiguous leads with prominent R-wave or R/S ratio>1.

Prior myocardial infarction: *Q-wave* in V2-V3 ≥0·02 sec or QS complex in V2-V3. Q-wave ≥0·03 sec and ≥1 mV deep in I, II, aVL, aF, or V4-V6 in any two leads of a contiguous lead grouping.

3. **Revascularisation**
Invasive revascularisation regardless of indication.
i. PCI with balloon or stent;
ii. CABG.

4. **TIA and stroke**
The distinction between a TIA and an ischaemic stroke is the presence of infarction. Duration of symptoms for 24 h is interpreted as presence of infarction, even when evidence is absent on imaging.

i. TIA

Transient ischaemic attack is defined as a transient (within 24 h) episode of focal neurological dysfunction caused by brain, spinal cord, or retinal ischaemia without acute infarction.

ii. Ischaemic stroke
Ischaemic stroke is defined as an acute episode of focal cerebral, spinal, or retinal dysfunction caused by infarction of the central neurons system.

iii. Haemorrhagic stroke
Acute episode of focal or global cerebral or spinal dysfunction caused by spontaneous intracerebral haemorrhage (excluding subarachnoid haemorrhage).

iv. Undetermined
Acute episode of focal or global cerebral, spinal, or retinal dysfunction caused by presumed infarction or haemorrhage, but with insufficient information to allow categorisation (i.e., imaging not performed).

STROBE Statement—checklist of items that should be included in reports of observational studies

	Item No.	Recommendation	Page No.	Relevant text from manuscript
**Title and abstract**	1	(*a*) Indicate the study's design with a commonly used term in the title or the abstract	1	Abstract
		(*b*) Provide in the abstract an informative and balanced summary of what was done and what was found	1	Abstract
**Introduction**				
Background/rationale	2	Explain the scientific background and rationale for the investigation being reported	2	Introduction
Objectives	3	State specific objectives, including any prespecified hypotheses	2	Introduction
**Methods**				
Study design	4	Present key elements of study design early in the paper	2-4	Methods
Setting	5	Describe the setting, locations, and relevant dates, including periods of recruitment, exposure, follow-up, and data collection	2-4	Study population, data collection
Participants	6	(*a*) *Cohort study*—Give the eligibility criteria, and the sources and methods of selection of participants. Describe methods of follow-up<br>*Case-control study*—Give the eligibility criteria, and the sources and methods of case ascertainment and control selection. Give the rationale for the choice of cases and controls<br>*Cross-sectional study*—Give the eligibility criteria, and the sources and methods of selection of participants	2-4	Methods
		(*b*) *Cohort study*—For matched studies, give matching criteria and number of exposed and unexposed<br>*Case-control study*—For matched studies, give matching criteria and the number of controls per case	n/a	

Variables	7	Clearly define all outcomes, exposures, predictors, potential confounders, and effect modifiers. Give diagnostic criteria, if applicable	2-4	End points
Data sources/ measurement	8*	For each variable of interest, give sources of data and details of methods of assessment (measurement). Describe comparability of assessment methods if there is more than one group	2-4	Data collection
Bias	9	Describe any efforts to address potential sources of bias	8	Strenghts and limitations
Study size	10	Explain how the study size was arrived at	2-3	Study population, figure 1

Continued on next page

Quantitative variables	11	Explain how quantitative variables were handled in the analyses. If applicable, describe which groupings were chosen and why	2-4	Methods
Statistical methods	12	(*a*) Describe all statistical methods, including those used to control for confounding	4	Statistical analysis
		(*b*) Describe any methods used to examine subgroups and interactions	4	Statistical analysis
		(*c*) Explain how missing data were addressed	4	Statistical analysis
		(*d*) *Cohort study*—If applicable, explain how loss to follow-up was addressed<br>*Case-control study*—If applicable, explain how matching of cases and controls was addressed<br>*Cross-sectional study*—If applicable, describe analytical methods taking account of sampling strategy	3-4	Data collection
		(*e*) Describe any sensitivity analyses		
**Results**				
Participants	13*	(a) Report numbers of individuals at each stage of study—eg numbers potentially eligible, examined for eligibility, confirmed eligible, included in the study, completing follow-up, and analysed		Figure 1
		(b) Give reasons for non-participation at each stage		Figure 1
		(c) Consider use of a flow diagram		Figure 1
Descriptive data	14*	(a) Give characteristics of study participants (eg demographic, clinical, social) and information on exposures and potential confounders		Table 1
		(b) Indicate number of participants with missing data for each variable of interest		Table 1
		(c) *Cohort study*—Summarise follow-up time (eg, average and total amount)	5	Results
Outcome data	15*	*Cohort study*—Report numbers of outcome events or summary measures over time	5	Results
		Case-control study—Report numbers in each exposure category, or summary measures of exposure		

		Cross-sectional study—Report numbers of outcome events or summary measures		
Main results	16	(*a*) Give unadjusted estimates and, if applicable, confounder-adjusted estimates and their precision (eg, 95% confidence interval). Make clear which confounders were adjusted for and why they were included	4-6	Statistical analysis and results
		(*b*) Report category boundaries when continuous variables were categorized		
		(*c*) If relevant, consider translating estimates of relative risk into absolute risk for a meaningful time period		

Continued on next page

Other analyses	17	Report other analyses done—eg analyses of subgroups and interactions, and sensitivity analyses	5-6	Results
**Discussion**				
Key results	18	Summarise key results with reference to study objectives	6	Discussion
Limitations	19	Discuss limitations of the study, taking into account sources of potential bias or imprecision. Discuss both direction and magnitude of any potential bias	8	Strength and limitations
Interpretation	20	Give a cautious overall interpretation of results considering objectives, limitations, multiplicity of analyses, results from similar studies, and other relevant evidence	6-8	Discussion
Generalisability	21	Discuss the generalisability (external validity) of the study results	6-8	Discussion
**Other information**				
Funding	22	Give the source of funding and the role of the funders for the present study and, if applicable, for the original study on which the present article is based	12	Source of funding

*Give information separately for cases and controls in case-control studies and, if applicable, for exposed and unexposed groups in cohort and cross-sectional studies.

Note: An Explanation and Elaboration article discusses each checklist item and gives methodological background and published examples of transparent reporting. The STROBE checklist is best used in conjunction with this article (freely available on the Web sites of PLoS Medicine at http://www.plosmedicine.org/, Annals of Internal Medicine at http://www.annals.org/, and Epidemiology at http://www.epidem.com/). Information on the STROBE Initiative is available at www.strobe-statement.org.
